# Successful treatment with carfilzomib and dexamethasone for relapsed/refractory POEMS syndrome: a case report and review of literature

**DOI:** 10.3389/fonc.2025.1570981

**Published:** 2025-06-06

**Authors:** Shuang Yu, Xiangxin Li, Jun Peng, Luqun Wang, Hao Li

**Affiliations:** Department of Hematology, Qilu Hospital, Cheeloo College of Medicine, Shandong University, Jinan, China

**Keywords:** POEMS syndrome, carfilzomib, VEGF, adverse events, peripheral neuropathy

## Abstract

**Background:**

POEMS syndrome is a rare multisystem disease secondary to plasma cell neoplasm. Due to its rarity, there are no internationally agreed treatment standards, with very limited data to guide management in the relapse setting.

**Case presentation:**

We describe a 51-year-old woman with initially presented with fatigue, anorexia, nausea, abdominal distension, and edema of the face and both lower limbs, who was diagnosed with POEMS syndrome accompanied with Raynaud’s phenomenon and cardiac involvement. After multiple lines of treatment, including bortezomib, cyclophosphamide, and dexamethasone (VCD), ixazomib, and daratumumab along with dexamethasone (DD), her clinical and laboratory features, and cardiovascular system continued to deteriorate. Then we started carfilzomib and dexamethasone, and the patient achieved a complete response. She did not develop significant cardiac toxicity and peripheral neuropathy. A total of 4 cycles of carfilzomib and dexamethasone were administered monthly, followed by autologous stem cell transplantation (ASCT). After 4 months of follow-up, a complete remission persists, and no significant complications were observed.

**Conclusion:**

We report on the first case of relapsed/refractory POEMS syndrome who received carfilzomib and dexamethasone, and achieved very good remission. Carfilzomib may be a safe and effective treatment option for patients with relapsed/refractory POEMS syndrome.

## 1 Introduction

POEMS (polyneuropathy, organomegaly, endocrinopathy, monoclonal protein, skin changes) syndrome is a rare paraneoplastic disorder characterized by clonal plasmic cell proliferation and multiple organ involvement (1). Due to its low incidence and the insidious onset of the variability of clinical manifestations, diagnosis of POEMS syndrome is often difficult, leading to misdiagnoses and delayed treatment. Delayed diagnosis may lead to organ dysfunction and subsequent adverse outcomes (2). The 5-year survival rate of patients with POEMS syndrome is 60% (3), and the continuous deterioration of neuropathy is a common cause of death for POEMS syndrome.

The treatment of POEMS syndrome essentially aims to eradicate underlying clonal plasma cells. A therapeutic challenge that we have in directing treatment selection for POEMS is the lack of randomized clinical trials due to the rarity of the disorder. For POEMS syndrome, no standard consensus guidelines of care are established, and treatment decisions for POEMS syndrome are mainly based on case serials and case reports (4, 5). In the relapse setting, data are scant with a range of agents, whose comprehensive profile of tolerability, toxicity, and responses is unclear (6). Carfilzomib, a new selective inhibitor of the chymotrypsin-like proteasome, showed remarkable activity in MM and amyloidosis by forming an irreversible, highly selective complex with proteasome via a unique mechanism (7–9). Notably, carfilzomib may possess advantages over first-generation agents, including a critical consideration given the preexisting peripheral neurological in POEMS syndrome. To date, data about the use of carfilzomib in POEMS syndrome are scarcely.

Here, we are the first to describe one case of a 51-year-old woman patient with relapsed/refractory POEMS syndrome successfully treated with carfilzomib and dexamethasone followed by autologous stem cell transplantation (ASCT). Currently, the patient is in a persistent remission state.

## 2 Case report

A 51-year-old woman presented to the Emergency Department on August 14, 2020, with a 10-day history of fatigue, anorexia, nausea, abdominal distension, and edema of the face and both lower limbs. She had a previous history of discoloration of hands in cold temperature, dry mouth, joint pain, renal insufficiency with symptoms of progressive edema and abdominal discomfort for 2 years. She had no history of smoking, drinking alcohol or substance usage; no known drug allergies; and no family history of hypertension, diabetes, kidney, or autoimmune diseases. On physical examination, the temperature was 36.1°C, pulse rate was 88 beats/min, blood pressure was 133/89 mmHg, and respiratory rate was 21 breaths/min. She appeared acutely ill and weak, with pale palpebral conjunctiva, skin hyperpigmentation, enlarged lymph nodes in the left side of the retro auricular, neck, and groin, the biggest of which was 2 cm × 3 cm in size, distended abdomen, obvious pitting edema in the lower limbs, and reduced superficial sensation in the distal extremities. No hepatosplenomegaly was observed and the remainder of the physical examination was normal.

The white cell count was 4.6× 10^9/^L. The hemoglobin level was 79 g/L, mean corpuscular volume 79.9 fl, mean hemoglobin content 26.1 pg, and platelet count 457× 10^9/^L. The erythrocyte sedimentation rate level was 73 mm/h. The plasma D-dimer level was 0.80 μg/ml and the fibrinogen degradation product level was 4.51 μg/ml. The blood urea nitrogen level was 8.61 mmol/L and the serum creatinine level was 223 μmol/L. The albumin level was 32.1 g/L and the uric acid level was 529 mmol/L. The serum iron level was 8.19 μmol/L (normal range, 9 to 27 μmol/L), the total iron-binding capacity was 29.19 μmol/L (normal range, 54 to 77 μmol/L), and the ferritin level was 442.5 ng/ml. Levels of aminotransferases, bilirubin, electrolytes, lactate dehydrogenase, vitamin B12, and folate were within normal range. Antibody tests for human immunodeficiency, Epstein-Barr virus, and cytomegalovirus were negative. Serum immunofixation electrophoresis showed a monoclonal protein of 2.46 g/L for IgG λ type. The level of free triiodothyronine was 1.90 pmol/L (normal range, 3.5–6.8 pmol/L). The levels of immunoglobulin, IgG4 and gonadal hormone were normal. The serum level of VEGF was 5034 ng/L (normal range, 0–160 ng/L). Tests for antinuclear antibodies, anti-double-stranded DNA, anti-RO, anti-Smith, and ANCA were normal.

**Figure 1:**
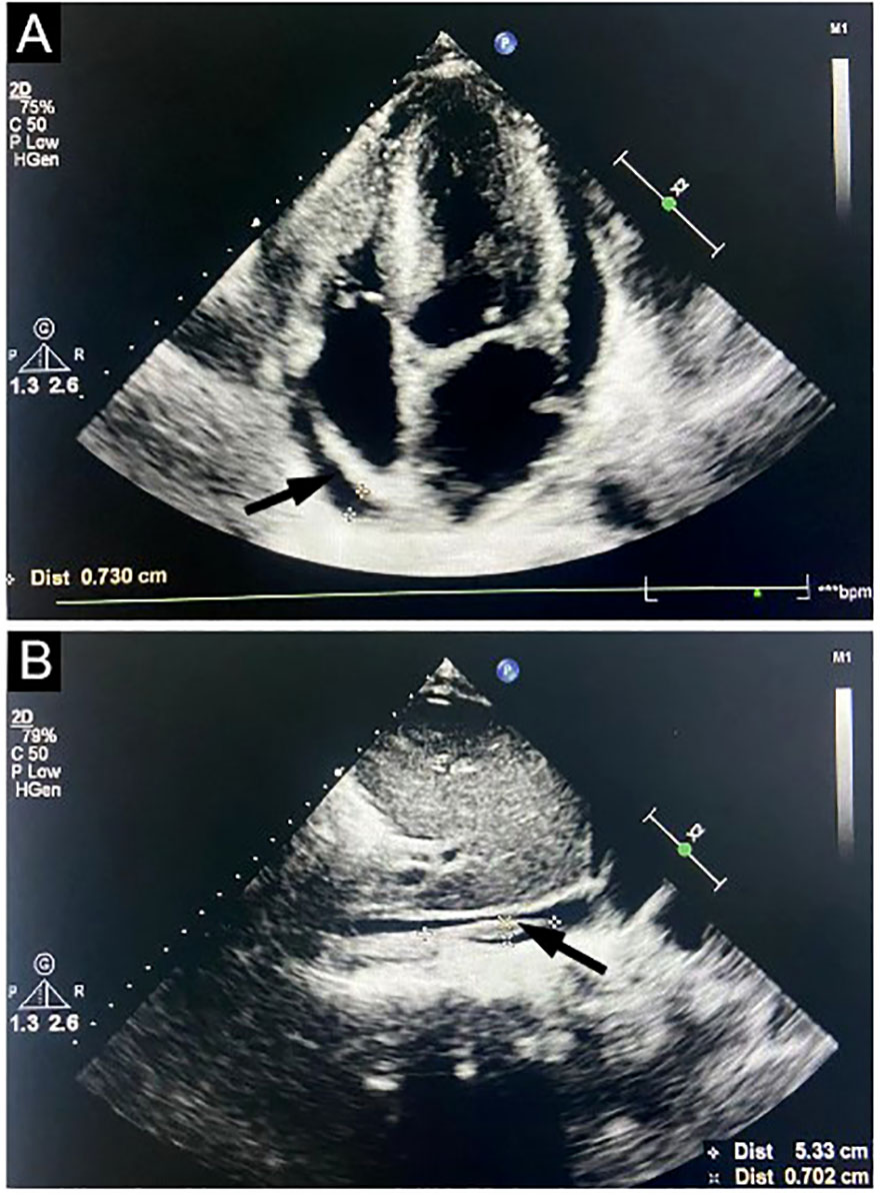
Echocardiography study. Doppler ultrasound images targeting the heart show left ventricular hypertrophy, thickened ventricular septum, enlargement of the left atrium, pericardial effusion (**A**, arrow), and thrombosis of inferior vena cava near the right atrium entrance (**B**, arrow).

**Figure 2:**
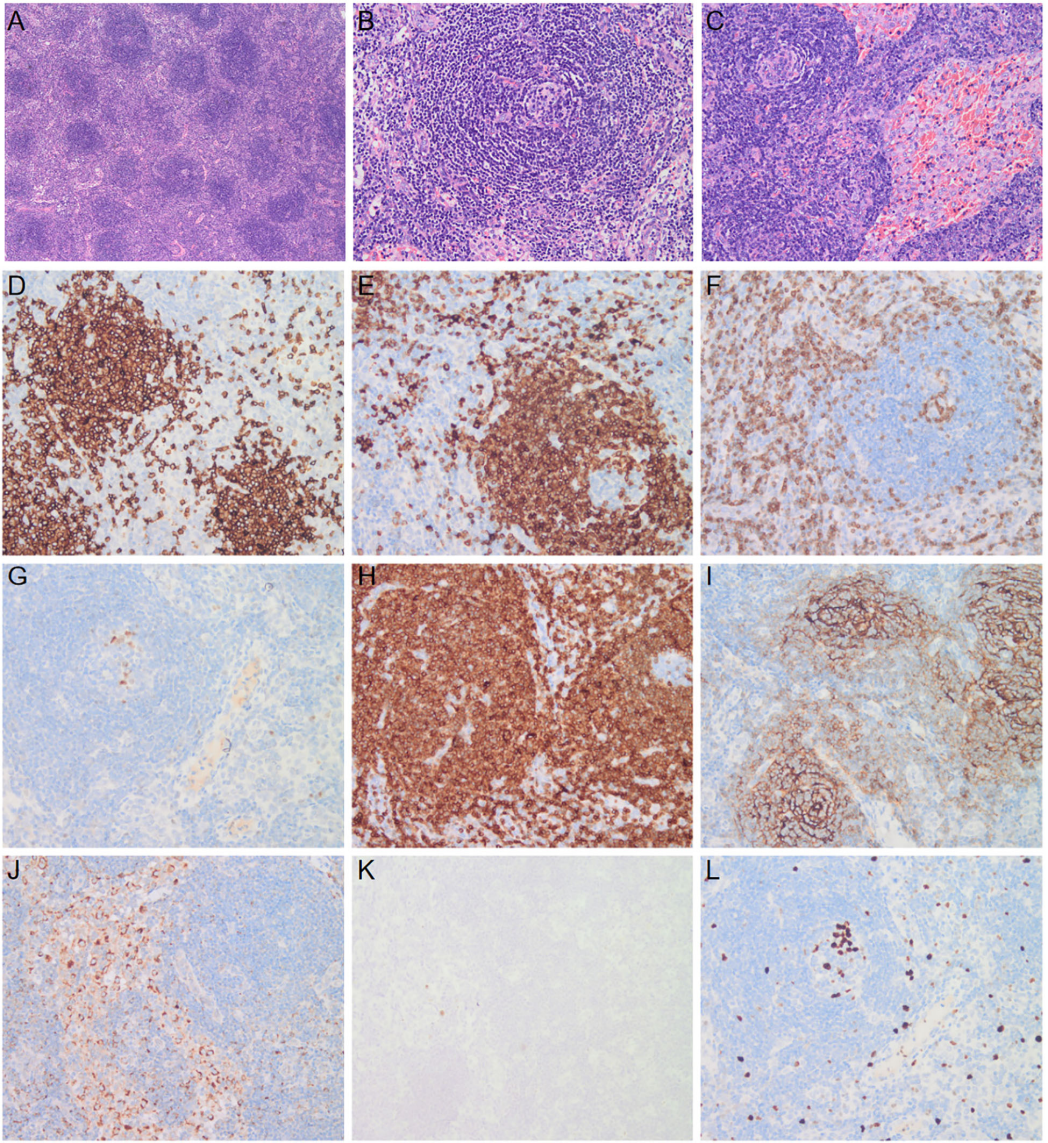
Biopsy specimen of lymph node. A biopsy specimen of the lymph node was obtained. Hematoxylin and eosin staining shows follicular hyperplasia and atrophic germinal center (**A**, ×4), vessels with hyaline changes (**B**, ×20), and lymph node parenchyma showing lymphatic sinus dilatation and sinus tissue cell hyperplasia (**C**, ×20). Immunohistochemical staining shows cell membrane and cytoplasm expression of CD20 **(D)**, CD79a **(E)**, CD3 **(F)**, Bcl6 **(G)**, Bcl2 **(H)**, CD21 **(I)**, and CD68 **(J)**, ×20. HHV8 staining is negative **(K)**, and the positive rate of Ki-67 was 10% **(L)**, ×20.

Bone marrow cytomorphology revealed that bone marrow hyperplasia was active, with no pathological plasma cells, and biopsy revealed few plasma cells were scattered. The congo red in BM was negative. Flow cytometry showed plasma cells had no phenotypic abnormalities. Echocardiography showed left ventricular hypertrophy, thickened ventricular septum (12 mm), enlargement of the left atrium, pericardial effusion, and thrombosis of the inferior vena cava near the entrance to the right atrium (**Figure 1**). Electromyography revealed polyneuropathy with demyelinating lesions of the peripheral nerves in both the upper and lower limbs. The non-contrast CT scan of the chest, abdomen, and pelvis revealed enlargement of mediastinal and bilateral axillary lymph nodes, a large amount of effusion in the pericardial, thoracic, abdominal, and pelvic cavity, along with swelling of the intestinal wall. Since the CT showed the presence of lymph nodes and polyserositis, a lymph node biopsy was performed to confirm the findings. A biopsy of the left axillary lymph node revealed follicular hyperplasia and atrophic germinal center, vessels with hyaline changes, and lymph node parenchyma showing lymphatic sinus dilatation and sinus tissue cell hyperplasia. Immunohistochemistry showed CD20 (focal +), CD79a (focal +), CD3 (T-region +), Bcl6 (germinal center +), CD10 (-), Bcl2 (-), CD21 (follicle dendritic cell +), CyclinD1(-), HHV8 (-), CD68 (common tissue cells +), and Ki-67 (10% +) (**Figure 2**).

**Figure 3:**
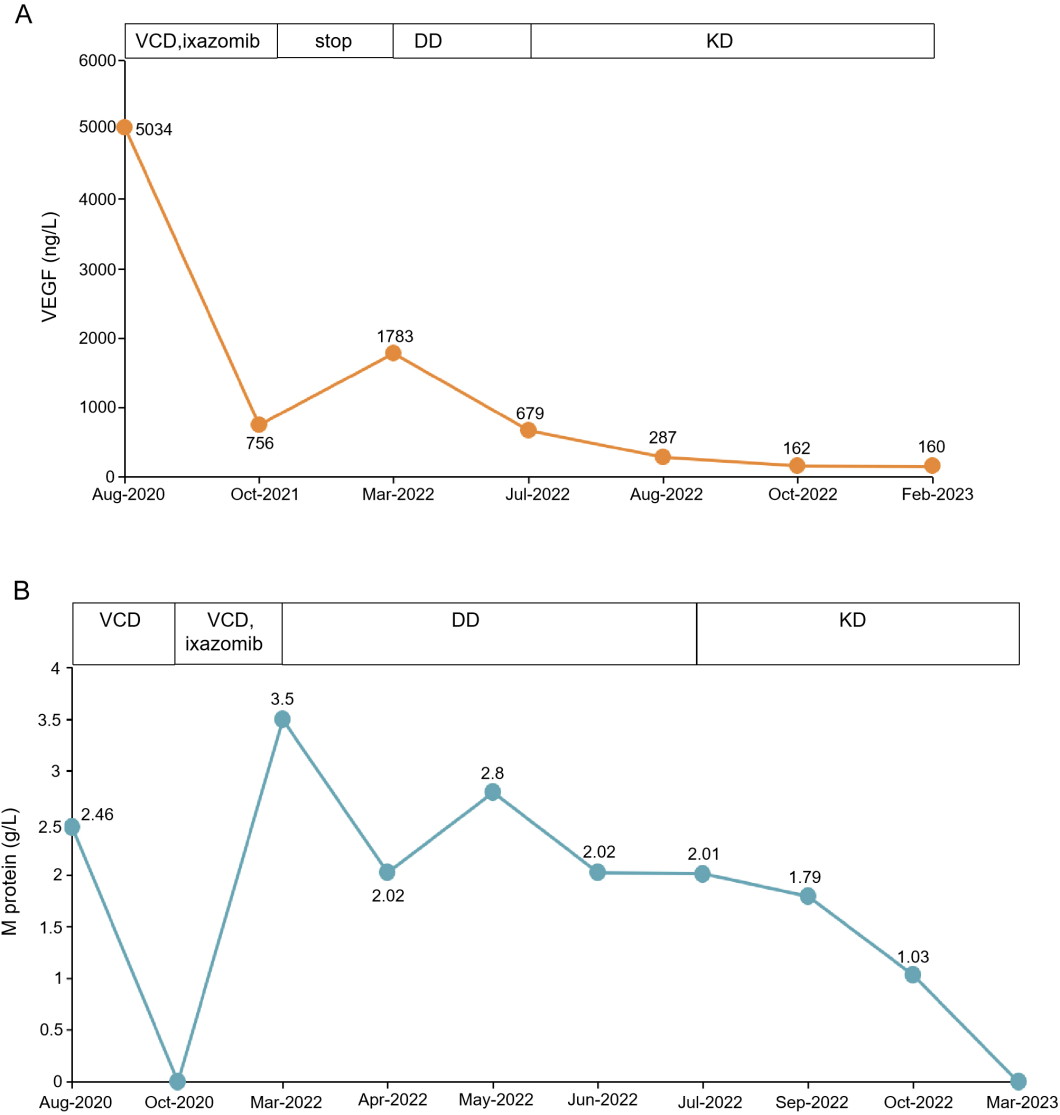
Clinical results of KD treatment. **(A)** shows the VEGF level before and after KD treatment. **(B)** shows the changes in M protein before and after treatment. VCD, bortezomib, cyclophosphamide and dexamethasone; KD, carfilzomib and dexamethasone; DD, daratumumab along with dexamethasone.

With all these findings, the patient was diagnosed with POEMS syndrome on August 29, 2020. After written informed consent, the patient was started on a 21-day cycle of BCD therapy (bortezomib, 1.3 mg/m^2^ subcutaneously on days 1, 4, 8, and 11; oral cyclophosphamide, 200 mg daily on days 1, 8, and 15; dexamethasone, 10 mg daily on days 1, 2, 4, 5, 8, 9, 11, and 12). Following the first course of treatment, the patient showed a remarkable improvement in her ascites, edema, and nerve symptoms. Two months later, the serum immunofixation electrophoresis, along with serum and urine light chain became negative, the platelet count and free triiodothyronine normalized, the hemoglobin level increased to 8.9 g/dL, the serum creatinine decreased to 138 μmol/L, and the thoracic and abdominal effusions significantly reduced. Subsequently, after completing 6 cycles of the BCD regimen, the patient received maintenance therapy of 10 cycles of oral ixazomib. The patient discontinued treatment once serum VEGF levels decreased to 756 ng/L. Three months later, the patient started complaining of fatigue, abdominal distension, and edema of the lower limbs. Biochemical examinations showed a relapse of POEMS syndrome with a total M protein level of 3.5 g/L and an increase in serum VEGF level (1783 ng/L), serum creatinine level (170 μmol/L), and prolactin level (559.70 uIU per ml). The echocardiogram showed invasive cardiomyopathy, pericardial effusion, and pulmonary hypertension. The CT scan revealed enlarged mediastinal, axillary, and retroperitoneal lymph nodes, along with increased effusion in the abdominal and pelvic cavity. The patient was administered daratumumab 16 mg/kg once weekly for 8 weeks along with dexamethasone, followed by four additional doses of daratumumab given fortnightly. One month later, the M protein level decreased to 2.8 g/L. However, the CT image showed more enlarged lymph nodes and polyserositis, and the echocardiogram revealed an incrassated ventricular septum (13 mm). The patient showed an invasive cardiomyopathy and pulmonary hypertension suggestive of cardiac toxicity secondary to the POEMS syndrome. Though the level of M protein decreased, the cardiovascular system continued to deteriorate after the daratumumab treatment. The ventricular septum was further thickened, which suggested a possibility of cardiac amyloidosis. The patient was recommended cardiac biopsy, for which she refused. After 2 months, the patient’s symptoms persisted. The M protein level was 2.01 g/L, the serum VEGF level was 679 ng/L, and serum creatinine increased to 179 mmol/L. Thereafter, she was treated with a 28-day cycle of carfilzomib (20/27 mg/m^2^ on days 1, 2, 8, 9, 15, and 16) and dexamethasone (20 mg on days 1, 2, 8, 9, 15, and 16) (KD). Two months later, biochemical evaluation of her IgG-λ showed a decreasing trend with normalization of VEGF level. Her peripheral polyneuropathy markedly improved and gonad functions became normal. An echocardiography examination showed a significant improvement in the cardiac system. The patient had demonstrated a rapid hematological response, along with achieving a complete remission (CR) in VEGF response (**Figure 3**). ASCT was performed after 4 cycles of KD regimen. At 4-month post-transplantation follow-up, serum VEGF remained normal and M protein turned negative. All clinical symptoms had disappeared, and all test results were improved. The thickness of ventricular septum decreased to 11 mm, and the invasive myocardial lesion and pulmonary hypertension disappeared. The overall neuropathy limitation scale (ONLS) score of the patients decreased from 4 at diagnosis to 0 at the last follow-up.

## 3 Discussion

The POEMS syndrome is a rare multisystem paraneoplastic disorder, characterized by the production of pro-inflammatory cytokines and angiogenic mediators (10). POEMS syndrome is underdiagnosed, owing to its low incidence and the insidious onset of the variable clinical manifestations. Currently, its diagnostic criteria are based upon Dispenzieri diagnosis, which needs the presence of 2 mandatory major criteria (polyneuropathy and monoclonal plasma cell disorder), at least 1 of the 3 other major criteria (sclerotic bone lesions, Castleman disease, and elevated VEGF level), and at least 1 of the 6 minor criteria (organomegaly, edema, endocrinopathy, skin changes, papilledema, thrombocytosis, or polycythemia).

In retrospect, there were many clues for diagnosing POEMS syndrome. The patient’s electromyography revealed peripheral neuropathy and the myeloma panel suggested a monoclonal plasma proliferative disorder. The patient was primitively diagnosed with Raynaud syndrome due to the initial manifestation of Raynaud’s phenomenon. Raynaud’s phenomenon encompasses other manifestations of POEMS syndrome and is frequently overlooked and misdiagnosed. To date, only two reports describe the prevalence of Raynaud’s phenomenon in POEMS syndrome (11). The underlying mechanism may be that elevated VEGF increased vascular permeability and proliferation with microangiopathy. VEGF is a potent cytokine compared to other inflammatory neuropathies and is correlated with disease activity and treatment response (12). Markedly elevated VEGF was another significant clue in our patient. The classic histomorphologic finding of lymph nodes is Castleman disease. Furthermore, the patient met five minor criteria (organomegaly, edema, endocrinopathy, skin changes and thrombocytosis). Other additional criteria were also presented including anemia, renal dysfunction, thrombotic complications, and pulmonary hypertension.

The diagnostic criteria of POEMS syndrome do not include cardiovascular system damage, which however is reported in 3% to 6% of cases (13). Although congestive myocardial dysfunction, a common complication in patients with amyloidosis, is rare in POEMS syndrome, several cases of congestive heart failure associated with POEMS syndrome have been reported (13–16). These patients underwent a myocardial biopsy that failed to detect amyloid deposition in the myocardial tissues. Shanshan et al. have reported myocardial dysfunction as one of the factors in POEMS syndrome (17). The cardiovascular system in our patient was characterized by left ventricular hypertrophy, thickened ventricular septum, enlarged left atrium, pericardial effusion, and pulmonary hypertension. The changes in the heart structure and function of the patient might be POEMS syndrome-related, because she had no coronary artery stenosis and a history of cardiomyopathy, and her congo red in bone marrow is negative. The mechanism underlying cardiac dysfunction in patients with POEMS syndrome is unclear. The elevated VEGF was suspected to be responsible for cardiac dysfunction. Studies have shown that markedly high serum VEGF levels may predispose patients with POEMS to the development of cardiac dysfunction, which can be reverted by a reduction in the VEGF level after successful treatment of POEMS syndrome (18). One recent report mentioned that pulmonary hypertension may be associated with dysfunction of the left ventricle (19). Therefore, the diagnosis of cardiac amyloidosis in patients with POEMS syndrome is challenging, and it is important to perform closer examinations, including myocardial biopsy if one patient is suspected of amyloidosis.

Currently, there is a paucity of randomized controlled clinical trials and established standard guidelines for the treatment of POEMS syndrome due to its inherent characteristics. The principle for treatment of POEMS syndrome is focused on eradicating the underlying plasma cell clone and suppressing the secretion of pro-inflammatory and pro-angiogenic cytokines. Therapeutic approaches are mostly based on retrospective studies, targeting plasma cell agents typically used in multiple myeloma (MM) and solitary plasmacytoma, including melphalan, lenalidomide, bortezomib, and novel agents. Involved-field radiotherapy is of limited benefit in localized bony disease. Given the anti-angiogenic effect of immunomodulatory drugs, lenalidomide provides clinical benefits in POEMS syndrome, where neuropathy is the feature of the disease itself. Melphalan combined with dexamethasone showed efficacy with low toxicity in the treatment of patients newly diagnosed with POEMS syndrome. Bortezomib is also an effective treatment for POEMS syndrome; however, concerns about neurotoxicity have limited its use historically. The anti-CD38 monoclonal antibody, daratumumab, is another promising option, but with limited data to support its use. Anti-VEGF monoclonal antibodies such as bevacizumab have a theoretical rationale but are not used because of their lack of efficacy and adverse effects leading to increased mortality in some cases (20).

As few randomized controlled trials in POEMS syndrome, there are no formal standards on transplant eligibility for this disease. Unlike MM, POEMS patients might not receive ASCT upfront even if they don’t need induction treatment. Historically, it likely relates to the low plasma cell burden in POEMS syndrome, which is sensitive to chemotherapy drugs, and many patients achieved durable responses with non-transplant therapies (6). Additionally, POEMS patients frequently present with severe peripheral neuropathy, capillary leak syndrome, and organomegaly, increasing the rate of transplant-related mortality, particularly with high-dose melphalan. In a large series study of 138 patients receiving ASCT, 32 subjects had induction prior to ASCT which enabled transplant eligibility. These patients were more likely to have respiratory compromise, ascites and renal impairment (2). Therefore, stabilizing organ functional status prior to transplant is more important than disease response. While upfront ASCT is occasionally employed in POEMS syndrome, severe end-organ dysfunction may prohibit patients from ASCT eligibility. The role of post-ASCT consolidation or maintenance chemotherapy in POEMS is unknown, and is not recommended at present. Further studies are needed to define optimal patient selection and transplant timing in this complex disorder.

Carfilzomib, a new selective inhibitor of the chymotrypsin-like proteasome activity, effectively inhibits proteasome activity by forming an irreversible, highly selective complex with proteasome via a unique mechanism. It has shown enhanced efficacy as a single agent, in combination with dexamethasone (KD), or with lenalidomide and dexamethasone (KRD) in MM and was subsequently used along with daratumumab and dexamethasone (DKD) (8, 9, 21). In addition, one recently published phase 2 clinical study demonstrated carfilzomib with pomalidomide and dexamethasone (KPD) regimens are highly effective in patients with relapsed/refractory MM (22). However, some MM cells have shown toxicity with intravenous administration of carfilzomib. The most frequent side effects were hematological and cardiovascular toxicities. Carfilzomib is also associated with a higher occurrence of cardiovascular adverse events (AEs) including hypertension, cardiac failure, arrhythmia, and thrombotic events (23). One recent meta-analysis of randomized phase 3 trials involving the use of carfilzomib for MM indicated 8.1% occurrence of heart failure in the carfilzomib arm compared with 3.4% in the control arm (24). The possible molecular mechanism can be explained by the upregulation of protein phosphatase (PP)-2A and subsequent inhibition of AMP-activated protein kinase-mediated autophagy (25). Currently, there is very few evidence for the efficacy and safety of carfilzomib in POEMS syndrome. Here, we present an extremely rare case of relapsed/refractory POEMS syndrome accompanied by Raynaud’s phenomenon and cardiac involvement, who was refractory to the upfront bortezomib, daratumumab, and dexamethasone. The patient was successfully treated with carfilzomib and dexamethasone. Her clinical symptoms improved, and gonad functions became normal. Her cardiac function, left ventricular hypertrophy, and pulmonary hypertension were gradually improved with carfilzomib treatment. Haematological response was rapid and excellent, with achieving a complete remission (CR) in the VEGF response. The patient did not develop significant cardiovascular AEs except for hypertension. No worsening peripheral neuropathy with carfilzomib was found.

This current study has two limitations. First, our work is a single-case report, and the treatment cycles of this case were relatively few. Another limitation is that the follow-up of this case was only for 4 months, so long-term outcomes were not yet available. Despite these limitations, the work successfully demonstrated the efficacy and safety of carfilzomib and dexamethasone in treating patients with POEMS syndrome. The response was remarkable, and no novel toxicities were appreciated, suggesting the combination of carfilzomib and dexamethasone emerges as a viable therapeutic alternative for POEMS syndrome. Nonetheless, larger-scale clinical trials are necessary to further substantiate the effectiveness of carfilzomib in POEMS syndrome.

## 4 Conclusion

Currently, there is no established treatment protocol for relapsed/refractory POEMS syndrome. Based on this case, the KD combination regimen shows remarkable effects without hematological and cardiac toxicity. This case highlights the potential of carfilzomib as a novel long-term therapeutic option for the management of POEMS syndrome and fills a gap in the use of carfilzomib for POEMS syndrome.

## Data Availability

The original contributions presented in the study are included in the article/supplementary material. Further inquiries can be directed to the corresponding authors.
